# Modulation of HIF-1α and STAT3 signaling contributes to anti-angiogenic effect of YC-1 in mice with liver fibrosis

**DOI:** 10.18632/oncotarget.21039

**Published:** 2017-09-16

**Authors:** Tzung-Yan Lee, Yann-Lii Leu, Chorng-Kai Wen

**Affiliations:** ^1^ Graduate Institute of Traditional Chinese Medicine, School of Chinese Medicine, College of Medicine, Chang Gung University, Taoyuan, Taiwan; ^2^ Department of Traditional Chinese Medicine, Chang Gung Memorial Hospital, Keelung, Taiwan; ^3^ Graduate Institute of Nature Products, College of Medicine, Chang Gung University, Taoyuan, Taiwan; ^4^ Center for Traditional Chinese Medicine, Chang Gung Memorial Hospital, Taoyuan, Taiwan

**Keywords:** YC-1, hypoxia-inducible factor-1α, angiogenesis, inflammation, fibrosis

## Abstract

Hypoxia has been shown to have a role in the pathogenesis of several forms of liver disease. The aim of the study was to evaluate the mechanisms of HIF-1α inhibitor, YC-1, during bile duct ligation (BDL)-induced liver fibrosis in mice. Liver fibrosis was induced in mice, and YC-1 was then given intraperitoneally (50 mg/kg) once daily following 5 days. Liver injuries mice that were treated with YC-1 showed improved inflammatory response and diminished angiogenesis and hepatic fibrosis. YC-1 treatment inhibited liver neutrophil infiltration, while a decreased in TNF-α signaling as well as macrophage aggregation. In addition, YC-1 downregulates iNOS and COX-2 levels by inhibiting the activation of NF-κB and STAT3 phosphorylation by negative regulation the expression of SOCS1 and SOCS3 signaling. On the other hand, YC-1 decreased angiogenesis, as shown by the downregulation of hypoxia-inducible cascade genes, i.e. VEGF. YC-1 treatment resulted in a significant decrease in hepatic fibrogenesis, α-SMA abundance, and TGF-βR1 expression as well as hypoxia were assessed using VEGFR1, vWF and HIF-1α immunostaining. These results suggest that multi-targeted therapies directed against angiogenesis, hypoxia, and fibrosis. Therefore, it may be suggested that YC-1 treatment may be a novel therapeutic agent for the treatment of liver disease.

## INTRODUCTION

Hepatic fibrosis is characterized by excessive deposition of extracellular matrix and collagen, and represents an intense process of tissue remodeling that is characterized by chronic inflammation, neoangiogenesis, and fibrogenesis [1]. Mechanisms controlling hepatic fibrosis involve different pathological processes. Two of them are major inducers of fibrogenic mediators and extracellular matrix molecules: a complex network of hypoxia and angiogenesis pathways.

Fibrotic tissue leads to decreased blood flow and oxygen delivery, thus becoming hypoxic. It had been reported that blood supply was impaired as a consequence of fibrosis which induced a hypoxic microenvironment in the cirrhotic livers [2–4]. In turn, liver fibrosis also promoted angiogenesis through inducing vascular endothelial growth factor (VEGF) from the activated hepatic stellate cells (HSCs) [5]. HIF is a master regulator of oxygen homeostasis and comprises two constitutively expressed subunits, including HIF-1α and HIF-1β. In hypoxia, the decreased protein proline hydroxylation causes HIF-1α to accumulate and translocate to the nucleus forming the transcriptionally competent HIF-1 that binds hypoxia response elements. Meanwhile, HIF-regulated genes comprise plasminogen activator inhibitor-1 (PAI-1), VEGF, tumor necrosis factor-α (TNF-α) and interleukin-1β (IL-1β) [6].

Angiogenesis, which has been shown to play an important role in the development of liver fibrosis [7–8], involves a tightly regulated network of cellular and molecular mechanisms that result in the formation of functional vessels. Mediators of inflammation have direct angiogenic activities, and angiogenesis, in turn, contributes to the amplification of the inflammatory response due to the expression of adhesion molecules and chemokines in the neovessels, which further promote inflammation status. Several mechanisms participate in the regulation of vascular endothelial growth factor (VEGF), a well-established angiogenic factor, including the recruitment of inflammatory cells, which then amplify angiogenesis via secretion of cytokines, such as TNF-ɑ, TGF-β, IL-1, and IL-6, that enhance angiogenesis either directly or via the upregulation of VEGF [9–10].

Proinflammatory mediators, as well as other hypoxic stimuli, can elicit an angiogenic response through the induction of hypoxia-inducible factor-1ɑ-dependent transcriptional activity, including VEGF production [11]. In addition, VEGF was recently found to have potent proinflammatory properties during hepatic fibrosis. In the liver, activated hepatic stellate cells (HSCs) express VEGF and VEGF receptors after treatment with carbon tetrachloride (CCl_4_) and may respond to hypoxia by expressing VEGF *in vitro* [12]. The effect of VEGF is mediated, in part, by its ability to facilitate intragraft mechanisms of leukocyte recruitment and to promote endothelial activation responses, including adhesion molecule and chemokine production [13]. Therefore, our aim was to target the network of hypoxia and angiogenesis signaling involved in hepatic fibrosis related to chronic inflammation. If it confirmed, hypoxia could be used as a potential prognostic marker of fibrosis progression and as a novel therapeutic target for the liver disorders.

YC-1, 3-(5-hydroxymethy-2-furyl)-1-benzylindazole, was developed as a potential therapeutic agent for circulation disorders by inhibiting platelet aggregation and vascular contraction [14–15]. Yeo et al indicted that YC-1 could block expression of HIF-1α and VEGF as well as halt the growth of xenograft tumor cells *in vivo* [16]. In addition, it had been reported that YC-1 suppressed LX-2 cell, a human hepatic stellate cell activation and induced it apoptosis through inhibiting α-smooth muscle actin (α-SMA) expression and promoting caspase-3 activity, respectively [17].

Therefore, this study was to determine if YC-1 causes the resistance to obstructive cholestatic liver injury following BDL. Three questions are major addressed: (i) are overall mice liver injury ameliorated by the administration of YC-1? (ii) is hepatic angiogenesis attenuated by YC-1 treatment; (iii) comparison of hepatic inflammatory response is regulated by YC-1. These data demonstrate that hypoxia inhibitor, YC-1, is a critical tool for the control of hepatic fibrogenesis during the inflammatory process.

## RESULTS

### YC-1 attenuates markers of hepatic injury

**Figure 1:**
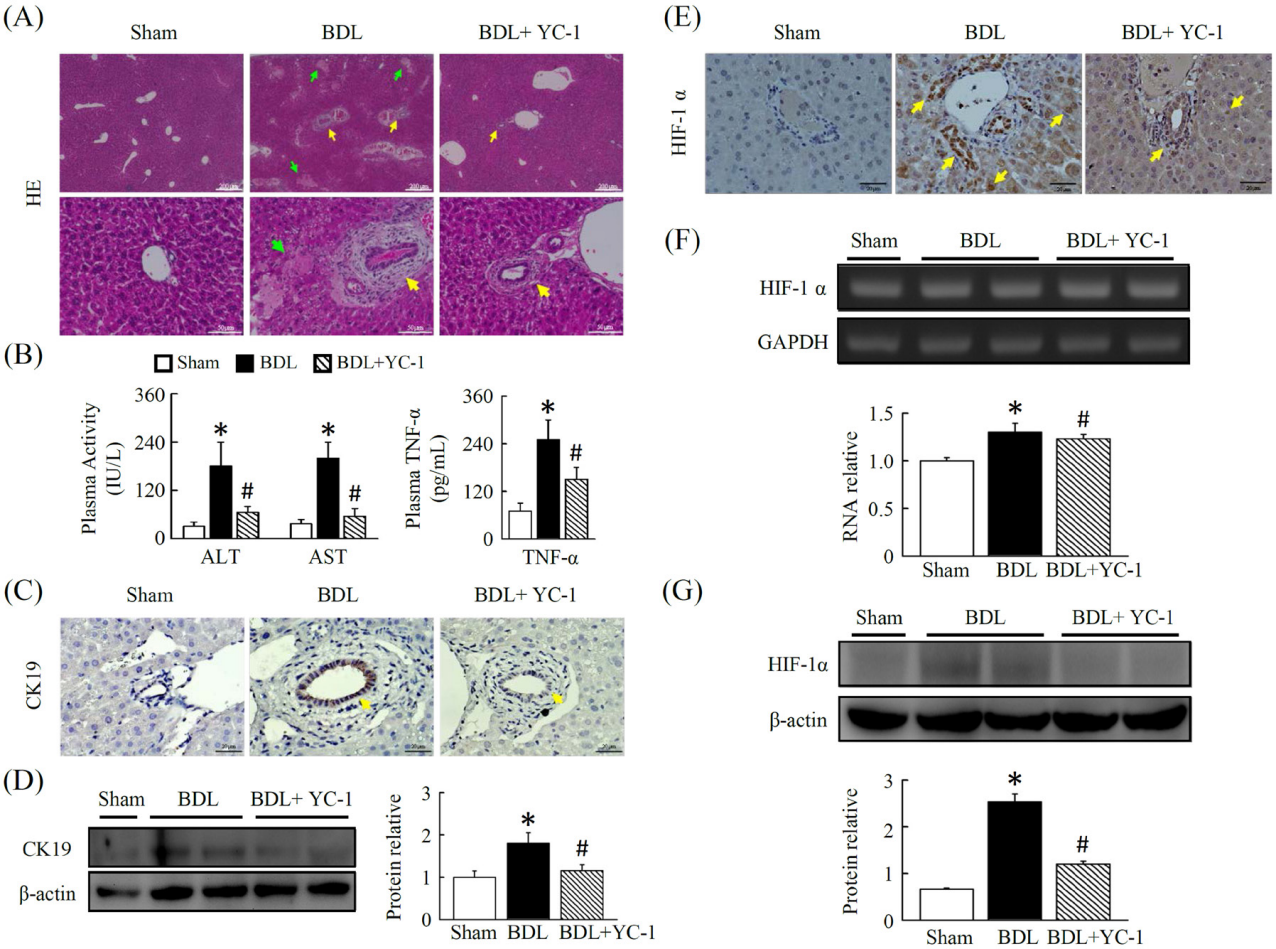
YC-1 attenuated cholestasis-induced liver injury in mice **(A)** Pronounced hepatoatrophy and necrosis is observed in livers after bile duct ligation. Mice treated with YC-1 (50 mg/kg) daily for 5 days show minor signs of hepatic necrosis in contrast to BDL mice. H&E staining (40× magnification) showing liver injury. **(B)** Mice were subjected to BDL, and liver injury were assessed at 5 days after surgery. ALT, AST and TNF-ɑ levels in plasma were increased to an extent in BDL mice, YC-1 attenuated BDL induced hepatic injury as analyzed with ALT, AST and TNF-ɑ levels. **(C, D)** Immunohistochemical identification of CK19 and protein expressions in Mice treated with YC-1 in contrast to BDL mice. **(E)** Immunohistochemical identification of HIF-1ɑ. **(F)** The gene expression induction of HIF-1ɑ in liver tissue exposed to BDL or with YC-1 for 5 days. **(G)** Western blotting of HIF-1ɑ. Densitometry analyses are represented as a relative ratio of HIF-1ɑ to β-actin. Bars represent mean±SEM from 5 samples per liver tissue type. (*p < 0.05 vs. sham; #p < 0.05 vs. BDL.)

The pronounced hepatoatrophy and coagulation necrosis after cholestasis (lighter areas, with marked inflammatory cell infiltration) was dramatically reduced and more focal in YC-1 treated mice (Figure 1A). Following bile duct ligation, a dramatic increase in liver enzyme activity was observed in BDL mice compared with sham mice. Treatment for 5 days with YC-1 attenuated the increase in serum transaminase compared to BDL mice (Figure 1B).

### YC-1 decreases BDL-induced CK19 and attenuates HIF-1ɑ activity

The expression of CK19 was negligible in control livers, as indicated by the low levels of CK19. Hepatic CK19 staining increased in BDL livers. These levels significantly decreased by YC-1 (50 mg/kg) treatment (Figure 1C, 1D). As shown in Figure 1E, hepatic HIF-1ɑ staining, HIF-1ɑ transcription levels (Figure 1F) as well as HIF-1ɑ protein levels (Figure 1G), was attenuated by YC-1 treatment compared to BDL mice, although the HIF-1α mRNA level did not present a significant difference among these groups.

### Effect of YC-1 on neutrophil and macrophage expression in BDL-treated mice

**Figure 2:**
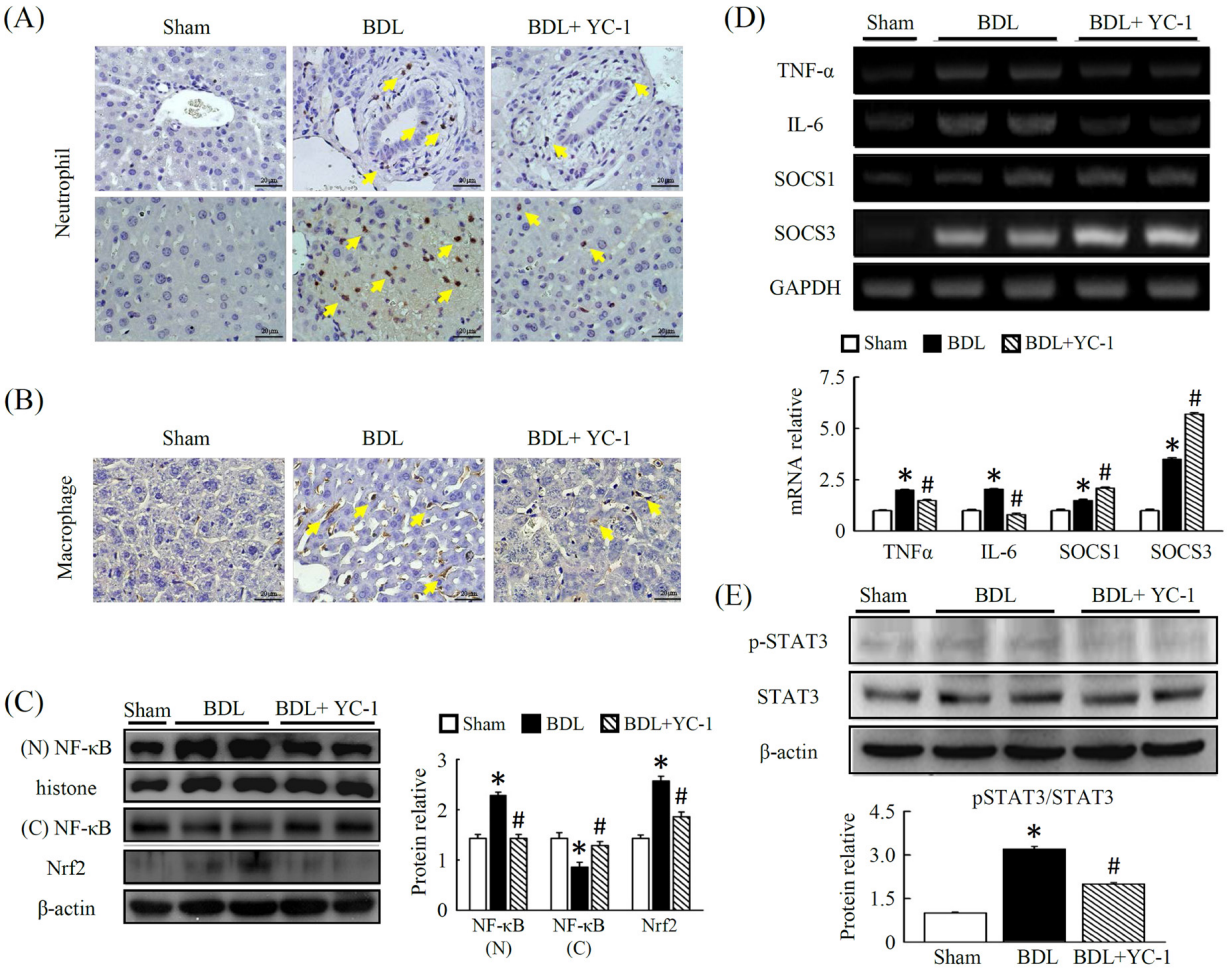
Effects of YC-1 on neutrophil and macrophage accumulation in the liver after BDL The extent of liver injury in mice given BDL were evaluated by antibody staining. Mice treated with YC-1 were also analyzed. Effective suppression is observed in the two strains (neutrophil and F4/80 staining as activated macrophage) treated with YC-1 in BDL mice **(A, B)**. The response of NF-κB, Nrf2 **(C)** and pSTAT3 **(E)**, protein activity in fibrotic mice treated with YC-1. **(D)** Hepatic tissue mRNA expression was determined by RT-PCR using GAPDH as the quantity control. The mRNA densitometry values were normalized to GAPDH levels. Data are presented as the mean ± SEM of at least three independent experiments. (*p < 0.05 vs. sham; #p < 0.05 vs. BDL.)

We performed immunohistochemical staining of liver infiltrating cells. When compared with control livers, macrophage (F4/80) expression, which is primarily localised in fibrotic tissue, increased in mice treated with BDL. Treatment with YC-1 diminished the number of cells per field of neutrophil (Figure 2A), and macrophage (F4/80) (Figure 2B) in the liver. In contrast, after BDL, there was an increase in NF-κB activity (Figure 2C), which was largely attenuated by YC-1 treatment, as determined using a nuclear activity assay in the treatment groups compared with the BDL group.

### YC-1 attenuates BDL-induced hepatic proinflammatory cytokine mRNA expression

As expected, YC-1 treatment decreased BDL-induced hepatic TNF-ɑ levels as well as STAT3 phosphorylation. In mice treated with YC-1, the expression of TNF-ɑ and IL-6 mRNAs decreased in liver tissues; in addition, the expression of these genes was attenuated in mice treated with YC-1 (Figure 2D). Moreover, Western blot analysis showed that hepatic levels of STAT3 phosphorylation in BDL mice were higher than sham-control mice; however, these phenomena were attenuated by treatment with YC-1 (Figure 2C).

### Effect of YC-1 on hepatic angiogenesis in mice with liver fibrosis

**Figure 3:**
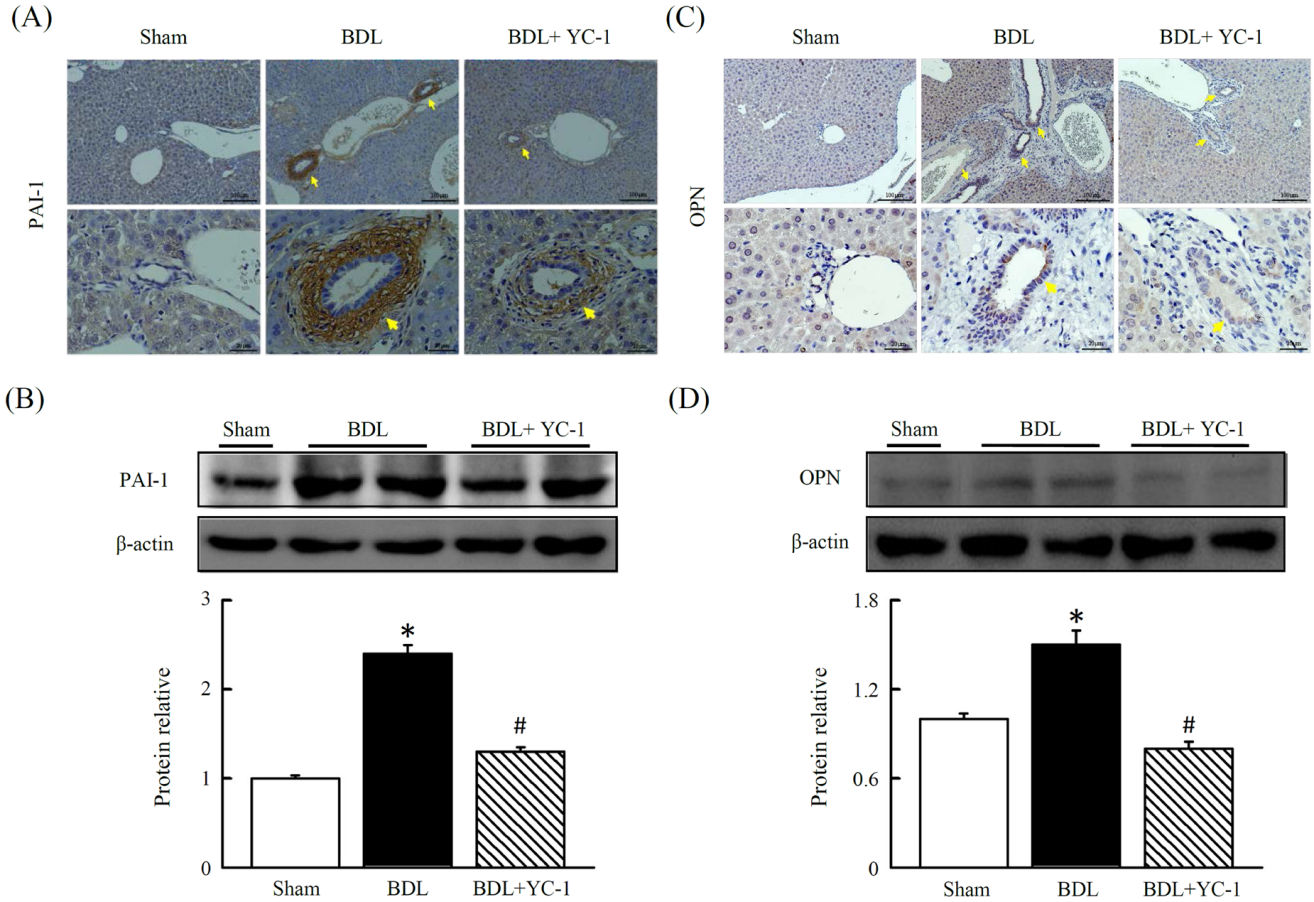
Effects of YC-1 on protein levels of plasminogen activator inhibitor-1 (PAI-1) and osteopontin (OPN) in the BDL mice Immunohistochemical analysis of liver sections from BDL or BDL plus YC-1-treated mice. The expression of PAI-1 **(A)** and OPN **(C)** were analyzed using PAI-1 or OPN antibodies, respectively. Immunoblot analysis of total liver lysates of BDL mice. BDL leads to a significant increase in PAI-1 **(B)** or OPN **(D)** protein levels, whereas YC-1 treatment shows more prominent changes. Equal amounts of lysate from sham or BDL mice were loaded in each lane and normalized against the control β-actin. The results are presented as the mean ± SEM from three independent experiments. (*p < 0.05 vs. sham; #p < 0.05 vs. BDL.)

**Figure 4:**
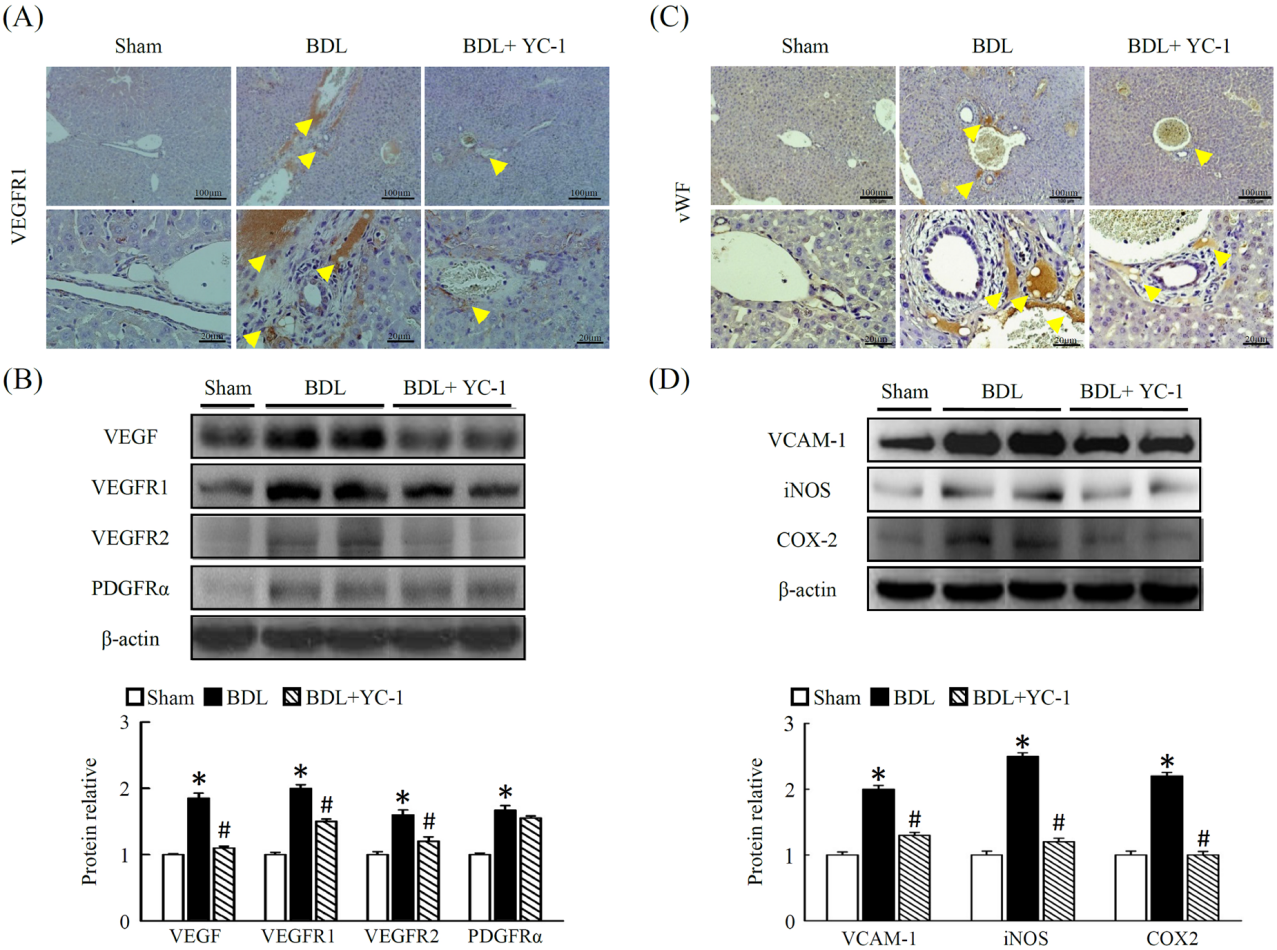
Effect of YC-1 on angiogenesis signaling in BDL mice **(A)** Livers were sectioned and staining with VEGFR1. VEGFR1-positive cells were observed adjacent to the central vein and portal triad. In contrast, after YC-1 treatment, the cells display a more ordered pattern and a lower VEGFR1-positive signals of pathologic sections (40× magnification). **(B)** Western blot analysis of the hepatic proteins VEGF, VEGFR1,2 and PDGFR-ɑ in the liver injury of mice. The densitometry values were normalized to β-actin. **(C)** Livers were sectioned and staining with vWF. In contrast, after YC-1 treatment, the cells display a more ordered pattern and a lower vWF-positive signals of pathologic sections (40× magnification). **(D)** Western blot analysis of the hepatic proteins VCAM-1, iNOS and COX-2 in the liver injury of mice. The densitometry values were normalized to β-actin. The results are presented as the mean ± SEM from three independent experiments. (*p < 0.05 vs. sham; #p < 0.05 vs. BDL.)

Mice conditioned with YC-1, in contrast to BDL mice, were resistant against BDL-induced hepatic hypoxia signalling, as evidenced by immunohistochemical staining of HIF-ɑ and vWF. The anti-vWF antibody was used to examine vascular proliferation. As expected, vWF staining was observed in the sinusoids and periportally adjacent to fibrous septa of injured tissue. The BDL group showed abundant histological criteria of hepatic HIF-ɑ and vWF (Figure 4C) that correlated with hepatic VEGFR1 (Figure 4). Livers with BDL-induced fibrosis revealed prominent angiogenesis staining within fibrotic septa. In addition, there was a marked increase in the abundance of PAI (Figure 3A, 3B), OPN (Figure 3C, 3D) and VEGFR1 (Figure 4A, 4B) in fibrotic livers compared with sham livers. Based on western blot and IHC analyses, treatment with YC-1 resulted in the downregulation of the HIF-1ɑ protein, and HIF-1ɑ-dependent signalling of VEGFR1.

VEGF is a key angiogenic factor, and its expression level influences the activation state of HSCs. The angiogenic phenotype of the stellate cells is possibly driven by increased expression of the VEGF and/or its receptors. As shown in Figure 4, hepatic VEGF and VEGFR-1 levels were higher in BDL mice compared with sham group. In contrast, both VEGF and VEGFR-1 levels were significantly lower in YC-1 treated mice. The expression of cell adhesion molecules in these newly formed blood vessels was examined by VCAM-1 expression. Fibrotic mice treat with YC-1 exhibited significantly reduced levels of VEGFR1 and VCAM (Figure 4D). The staining showed that inflammatory infiltrate signalling was mainly composed of angiogenesis and fibrosis symptom. Following YC-1 treatment that inhibition of angiogenesis, a significant reduction in macrophage and neutrphil infiltration was detected in fibrotic livers, which correlated with the diminution of the vasculature found in fibrotic septa.

### Effect of YC-1 treatment on hepatic fibrosis

**Figure 5:**
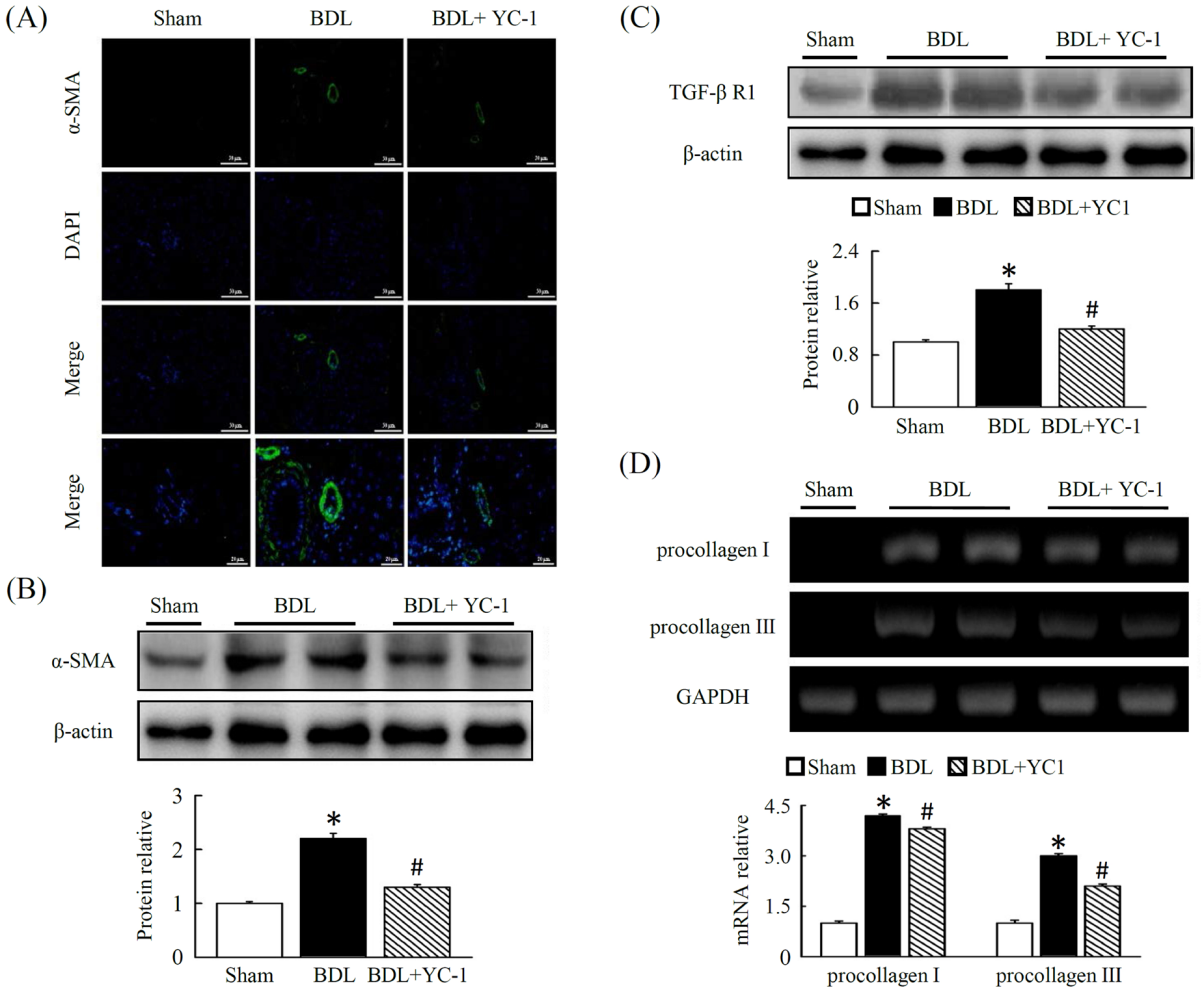
Fibrotic mice treated with YC-1 exhibited a significant reduction in hepatic levels of angiogenesis and symptom of fibrosis **(A)** Fluorescent immunostaining of liver sections of BDL mice. Nuclei were counterstained with DAPI (blue). The expression and localisation of ɑ-SMA (green), a marker of activated HSCs, were analysed using anti-ɑ-SMA antibodies, respectively. Mice overexpressing ɑ-SMA in the livers of BDL mice. Western blot analysis of hepatic ɑ-SMA **(B)**, TGF-βR1 **(C)** proteins in the livers of sham, BDL, and BDL plus YC-1-treated mice. The densitometry values were normalized to that of β-actin. Hepatic tissue procollagen I, III mRNA **(D)** expression were determined by RT-PCR using GAPDH as the internal control. The results are presented as the mean ± SEM from three independent experiments. (*p < 0.05 vs. sham; #p < 0.05 vs. BDL.)

Treatment with YC-1 inhibited irregular vascular patterning in the central/ periportal areas that were surrounded with fibrotic septa. In addition to improved liver angiogenesis, fibrosis was also quantified based on levels of hepatic ɑ–SMA density in fibrotic areas, strong collagen staining was observed around the portal tracks and fibrotic septa in the livers of BDL mice. In contrast, fibrotic mice treated with YC-1 showed a significant decrease in hepatic collagen. To study the underlying cellular events responsible for decreased liver fibrosis in YC-1 treated mice, we performed ɑ–SMA staining (Figure 5A) and western blot analysis (Figure 5B) of liver samples. Hepatic ɑ–SMA expression and protein levels were significantly reduced in YC-1 treated fibrotic mice compared with BDL group. We also performed a western blot analysis to determine if transforming growth factor (TGF)-β receptor 1 levels were affected (Figure 5C), in line with fibrosis area were identical, suggesting that suppression of HSCs activation also contributed to the antifibrotic effects of YC-1. In parallel, the procollagen-III mRNA level increased and decreased in the liver tissues of the BDL mice in the absence and presence of YC-1, respectively, although YC-1 did not inhibit the hepatic procollagen-I mRNA expression which induced by BDL (Figure 5D).

## DISCUSSION

The data presented here identifies YC-1 as a potential therapy for hepatic fibrosis that may simultaneously target multiple signaling pathways that influence both the excessive activation of the inflammatory response and hypoxia-induced angiogenesis.

The high energy consumption of hepatocytes renders them vulnerable to reduction in oxygen availability [18]. It was reported that HIF-1α is activated in the livers of mice with fibrosis induced by BDL [19]. Herein, the present study demonstrated that YC-1 administration improved liver fibrosis in mice, which was caused by a BDL through, at least in part decrease of HIF-1α-induced inflammatory cells infiltration, angiogenesis and bile duct epithelial cell proliferation. Of note, biliary epithelial cell proliferation is obvious elevated in liver after BDL treatment from the result of hepatic expression level of CK-19 [20]. Meanwhile, cirrhotic nodules are surrounded by a rim of CK-19-positive biliary cells in most intraseptal hepatocytes termed ductular reaction [21]. Herein, our current study showed that the increased liver fibrosis in the BDL mice was associated with an increase in bile duct epithelial CK-19 expression via up-regulation of hepatic HIF-1α level. Meanwhile, the observation that YC-1 treatment reduced hepatic CK-19 expression in BDL mice. This indicated that the hypoxia and its regulated transcription factor-HIF-1α played an important role in the mechanisms of unbalanced bile acids transporting of cholangiocytes in biliary epithelial damages following BDL.

This study revealed that hepatic fibrosis is accompanied not only by inflammatory response but also by extensive hepatic angiogenesis. The inflammatory response is regulated by the induction of hypoxia signalling as evidence with HIF-1ɑ, which is necessary for the recruitment of inflammatory cells to sites of inflammation. Previous study had shown that depletion of macrophages slowed the progression of fibrosis in rodent models [22]. Moreover, Copple et al suggested that deletion of HIF-1α in myeloid cells was responsible for the reduction of fibrosis in cholestatic mice, although plasma ALT level and TNF-α expression in myeloid cells remained unchanged [23]. Our results revealed that a significant decrease in plasma ALT, AST and TNF-α concentrations as well as in extensive accumulation of neutrophils and macrophages in liver tissues occurred in BDL mice following the administration of 50 mg/kg YC-1. The number of neutrophile-positive cells within the liver increased following BDL challenged, which coincided with the accumulation of activated F4/80-positive macrophages. In addition, cholestasis-induced liver injury is histopathologically characterised by the centrilobular necrosis and the excess infiltration of neutrophils. YC-1 treatment may downregulate the threshold for neutrophil-dependent hepatotoxicity in a BDL model of liver injury. Accordingly, we may speculate that neutrophils and macrophages in the liver might promote fibrosis by releasing profibrotic growth factors that stimulate HSCs to produce collagen after BDL challenge, and these phenomena were neutralized by YC-1.

However, we do not know whether YC-1 treatment further reduce nuclear HIF-1α level to bind hypoxia response elements in DNA from isolated Kupffer cells (KCs) against BDL-induced liver injury in our model. On the other hand, it was reported that the NF-κB activity is necessary for HIF-1α in the hypoxic liver [3, 24]. Meanwhile, the amount of VCAM had increased during hypoxia-reoxygenation in the BDL model. Our study is the first reported that HIF-1α is able to simultaneously modulate hepatic PAI-1 and VCAM levels after BDL challenge and speculated that NF-κB played an important role involving these mechanisms.

VEGF is a hypoxia-induced angiogenic factor in the vascular proliferation associated with wound healing and tumor growth. VEGF expression also correlates with angiogenesis and sinusoidal capillarisation associated with chronic liver diseases [25]. In addition, VEGF may also play a major role in directly affecting mice HSCs by stimulating the synthesis of collagen and inducing cellular proliferation [26]. Meanwhile, VEGF interacted with VEGFR-1 and VEGFR-2 that are selectively expressed by vascular endothelium to mediate its biologic effects [27]. It is noteworthy that activated HSCs secrete VEGF and angiopoietin-1 to promote angiogenesis in the model of murine liver fibrosis or exposure to leptin [28–29]. OPN exists in the bile duct epithelium of liver physiologically; however, liver pathological expression of OPN was occurred in KCs and HSCs in necrotic areas after carbon tetrachloride intoxication [30]. Moreover, coexpression of VEGF and OPN correlated with angiogenesis in patients with stage I lung adenocarcinoma [31]. On the other hand, vWF is an angiogenic-associated factor and had been enhanced in dimethylnitrosamine induced liver fibrosis rat model [32]. According to our findings, it might speculate that there is an interaction between VEGF and OPN to contribute to angiogenesis through enhancing vascular endothelial VEGFR-1 and VEGFR-2 in our BDL mice model, and significantly suppressed by YC-1. YC-1 was effective in reducing neovascularisation and other crucial parameters of fibrosis including the number of ɑ-SMA-positive cells present in liver tissue. As previously reported, the expression of VEGF and VEGFR1 is observed in liver tissues undergoing chronic wound healing [26, 29, 33–34]. The colocalisation data presented here highlight that the concomitant expression of ɑ-SMA, VEGFR1 and VCAM-1 is restricted to activated ɑ-SMA-positive cells localised at the leading edges of developing fibrotic septa and bridging septa. Taken together, these results suggest that YC-1 may be able to modulate multiple and concomitant processes, such as neoangiogenesis, inflammation and fibrogenesis. The link between angiogenesis and inflammatory infiltration in fibrosis livers suggests that YC-1 may interfere with the progression of chronic liver disease.

However, this study expands upon those findings in several significant ways. First, YC-1 treatment decreased the inflammatory response in fibrotic livers. This beneficial effect is likely due to a decrease in the number of hepatic neutrophil/macrophage infiltrations, which contributes to the downregulation of the proinflammatory cytokine cascade and cell proliferation. Second, YC-1 treatment significantly decreased angiogenesis in fibrotic mice, which may be due to the inhibition of HIF-1ɑ signaling pathways. Various biological functions and pharmacological actions of YC-1 have been contributed to anticancer activity effects by suppression of HIF-1α [16, 24, 35]. Xiao et al indicated that YC-1 derivatives might be potential agents for hepatic fibrosis therapy through suppressing activated LX-2 cell, a human HSC cell viability and inducing cell apoptosis [17].Therefore, it is likely that YC-1 decreased ɑ-SMA and extracellular matrix accumulation in livers may through the inhibition of HSCs via multiple mechanisms.

In conclusion, this study demonstrates that therapies that molecularly target hypoxia, inflammation, and angiogenesis together might be beneficial in the treatment of liver fibrosis.

## MATERIALS AND METHODS

### Materials

YC-1, ɑ-SMA and β-actin antibodies were from Sigma-Aldrich (St. Louis, MO); TNF-ɑ, and IL-6 kits were from R&D Systems (Minneapolis, MN); serum alanine aminotransferase kit and ECL were obtained from Amersham (Piscataway, NJ); anti-neutrophil antibody was from Abcam (Cambridge, MA); vWF antibody was from Thermo Scientific (Rockford, IL), F4/80 was from Biolegend; anti-HIF-1ɑ antibody was from Santa Cruz; VCAM-1 antibody was from Millipore (Billerica, MA); DAPI antibody was obtained from Invitrogen (Carlsbad, CA); anti-VEGFR1 antibody was from Epitomics (Burlingame, CA).

### Animal models and drug treatment

To induce obstructive cholestasis, 15 animals underwent bile duct ligation (BDL). BDL was performed under ketamine/xylazine anesthesia via a midline laparotomy. Using a stereoscopic microscope, the common bile duct was isolated and ligated carefully with a 6-0 silk suture. The laparotomy was then closed by a 5-0 running suture, and the animals were allowed to recover from anesthesia and operation for 12 hours. Sham-operated animals underwent an identical laparotomy and liver manipulation without BDL (sham, n = 5). Bile duct–ligated animals (n = 5), which received DMSO (0.01%) only, served as positive controls. To delineate the role of YC-1 in HIF-1ɑ activation during obstructive cholestasis, mice (n =7) were treated YC-1 daily for 5 days. YC-1 was dissolved in dimethyl sulfoxide (DMSO), which was then serially diluted in normal saline prior to use. All animal experimental protocols were approved by the Chang Gung University Animal Care and Use Committee (IACUC Approval No.: CGU14-046) in accordance with international standards of humane animal use.

### Biochemical measurement

Animals were sacrificed using CO2 inhalation. Blood was collected by intracardiac puncture and stored overnight (4°C). Serum was then collected and used to measure alanine aminotransferase and cytokines. Plasma ALT and AST analyses were performed using a commercially available diagnostic kit (Randox Laboratories, Antrim, UK). The plasma level of TNF-α was determined using a commercial ELISA kit (R&D system, Minneapolis, MN) according to the manufacturer’s instructions. Livers were harvested and weighed, then fixed and embedded for histopathological and immunohistochemical analysis. Some liver samples were snap-frozen in liquid nitrogen and stored at −80 °C until required.

### Histopathology assay and neutrophils measurement

Liver tissue was fixed in 10% formalin and then embedded in paraffin prior to being cut into 5 μm thick sections and stained with hematoxylin-eosin (H&E) or Masson’s trichrome. Sections were then examined under light microscopy by a pathologist. Sections were also used for histochemical localisation of esterase activity to identify neutrophils based on positive staining and morphology of nuclear segmentation.

### Immunohistochemistry and immunofluorescence staining

Paraffin sections were dewaxed, and rehydrated using xylene and successive ethanol baths. After extensive washes in phosphate-buffered saline (PBS), tissue sections were incubated with specific primary antibodies for 1 h at 37°C in a humidified atmosphere and then washed three times with PBS. For immunohistochemical studies, the following antibodies were used: ɑ-smooth muscle actin (ɑ-SMA) (1:100), neutrophil (1: 50), CK19 (1: 100), F4/80 (1: 75), HIF-1ɑ (1: 50), vWF (1: 100), VEGFR1 (1: 100). Samples were incubated with peroxidase- or fluorescently-compound-conjugated secondary antibodies for 1 h at 37°C followed by extensive washes in PBS. Fluorescent images were visualised using a Zeiss LSM 510 META confocal fluorescence microscope (Carl Zeiss, Jena, Germany). The number of activated HSCs in the liver was estimated using double immunofluorescence staining with the anti-ɑ-SMA and anti-desmin antibodies. Nuclear staining (blue fluorescence) was obtained by treating liver sections with 4,6-diamidino-2-phenylindole (DAPI)

### Western blot analysis

Liver tissue was lysed with protease inhibitors in distilled water, and a Bio-Rad Rapid Coomassie kit was used to determine total protein concentration. In all, 60 μg of protein was run on a 10% SDS-polyacrylamide gel and transferred to a polyvinylidene difluoride membrane. Immunoblotting was performed with various mouse or rabbit monoclonal or polyclonal antibodies, followed by incubation with the appropriate secondary antibodies coupled with horseradish peroxidase. The blot was developed using a chemiluminescence system (ECL; Amersham, Piscataway, NJ) according to the manufacturer’s instructions. The optical densities of the bands were measured with a Model GS-700 Imaging Densitometer (Bio-Rad, Hercules, CA). β-actin was used as a loading control.

### Reverse transcription-polymerase chain reaction (RT–PCR) assay

Total RNA was extracted from liver tissues using the guanidinium-phenol-chloroform method. Total RNA (5 μg) was reverse-transcribed as cDNA using RevertAid™ First Strand cDNA Synthesis Kit (Thermo Scientific, Waltham, MA) according to the manufacturer’s protocol. The single-stranded cDNA was amplified using Taq DNA polymerase kit (Thermo Scientific) and performed a set of denaturation at 95°C for 3 min and posterior to 30 cycles of amplification, a cycle contains at 95°C for 30 s, at 55°C for 30 s, and at 72°C for 30 s, then final extension was terminated at 72°C for 7 min. PCR products were separated by electrophoresis on 3% agarose gel and quantified by ImageQuant 5.2 software (Healthcare Bio-Sciences, Philadelphia, PA) software. The following primer sequences were used: HIF-1α, 5’-TCAAGTCAGCAACGTGGAAG-3’ (sense) and 5’-TATCGAGGCTGTGTCGACTG-3’ (antisense); Procollagen-I, 5’-TACTACCGGGCCGATGC-3’ (sense) and 5’-TCCTTGGGGTTCGGGCTGATGTA-3’ (antisense); Procollagen-III, 5’-CCCCTGGTCCCTGCTGTGG -3’ (sense) and 5’-GAGGCCCGGCTGGAAAGAA -3’ (antisense); GAPDH, 5’-CCCTTCATTGACCTCAACTACATGG -3’ (sense) and 5’-CATGGTGGTGAAGACGCCAG -3’ (antisense).

### Statistical analysis

The results are expressed as means ± SEM. Statistical analysis was performed using a one-way analysis of variance followed by a Student–Newman–Keuls’ multiple-range test. A p value of < 0.05 was considered statistically significant.

## References

[1] Friedman SL (2003). Liver fibrosis—from bench to bedside. J Hepatol.

[2] Kim KR, Moon HE, Kim KW (2002). Hypoxia-induced angiogenesis in human hepatocellular carcinoma. J Mol Med (Berl).

[3] Rosmorduc O, Housset C (2010). Hypoxia: a link between fibrogenesis, angiogenesis, and carcinogenesis in liver disease. Semin Liver Dis.

[4] Bozova S, Elpek GO (2007). Hypoxia-inducible factor-1alpha expression in experimental cirrhosis: correlation with vascular endothelial growth factor expression and angiogenesis. APMIS.

[5] Thabut D, Shah V (2010). Intrahepatic angiogenesis and sinusoidal remodeling in chronic liver disease: new targets for the treatment of portal hypertension?. J Hepatol.

[6] Murdoch C, Muthana M, Lewis CE (2005). Hypoxia regulates macrophage functions in inflammation. J Immunol.

[7] El-Assal ON, Yamanoi A, Soda Y, Yamaguchi M, Igarashi M, Yamamoto A, Nabika T, Nagasue N (1998). Clinical significance of microvessel density and vascular endothelial growth factor expression in hepatocellular carcinoma and surrounding liver: possible involvement of vascular endothelial growth factor in the angiogenesis of cirrhotic liver. Hepatology.

[8] Yoshiji H, Kuriyama S, Yoshii J, Ikenaka Y, Noguchi R, Hicklin DJ, Wu Y, Yanase K, Namisaki T, Yamazaki M, Tsujinoue H, Imazu H, Masaki T (2003). Vascular endothelial growth factor and receptor interaction is a prerequisite for murine hepatic fibrogenesis. Gut.

[9] Pertovaara L, Kaipainen A, Mustonen T, Orpana A, Ferrara N, Saksela O, Alitalo K (1994). Vascular endothelial growth factor is induced in response to transforming growth factor-beta in fibroblastic and epithelial cells. J Biol Chem.

[10] Cohen T, Nahari D, Cerem LW, Neufeld G, Levi BZ (1996). Interleukin 6 induces the expression of vascular endothelial growth factor. J Biol Chem.

[11] Richard DE, Berra E, Pouyssegur J (2000). Nonhypoxic pathway mediates the induction of hypoxia-inducible factor 1alpha in vascular smooth muscle cells. J Biol Chem.

[12] Ankoma-Sey V, Wang Y, Dai Z (2000). Hypoxic stimulation of vascular endothelial growth factor expression in activated rat hepatic stellate cells. Hepatology.

[13] Reinders ME, Sho M, Izawa A, Wang P, Mukhopadhyay D, Koss KE, Geehan CS, Luster AD, Sayegh MH, Briscoe DM (2003). Proinflammatory functions of vascular endothelial growth factor in alloimmunity. J Clin Invest.

[14] Teng CM, Wu CC, Ko FN, Lee FY, Kuo SC (1997). YC-1, a nitric oxide-independent activator of soluble guanylate cyclase, inhibits platelet-rich thrombosis in mice. Eur J Pharmacol.

[15] Galle J, Zabel U, Hubner U, Hatzelmann A, Wagner B, Wanner C, Schmidt HH (1999). Effects of the soluble guanylyl cyclase activator, YC-1, on vascular tone, cyclic GMP levels and phosphodiesterase activity. Br J Pharmacol.

[16] Yeo EJ, Chun YS, Cho YS, Kim J, Lee JC, Kim MS, Park JW (2003). YC-1: a potential anticancer drug targeting hypoxia-inducible factor 1. J Natl Cancer Inst.

[17] Xiao J, Jin C, Liu Z, Guo S, Zhang X, Zhou X, Wu X (2015). The design, synthesis, and biological evaluation of novel YC-1 derivatives as potent anti-hepatic fibrosis agents. Org Biomol Chem.

[18] Martin H, Sarsat JP, Lerche-Langrand C, Housset C, Balladur P, Toutain H, Albaladejo V (2002). Morphological and biochemical integrity of human liver slices in long-term culture: effects of oxygen tension. Cell Biol Toxicol.

[19] Moon JO, Welch TP, Gonzalez FJ, Copple BL (2009). Reduced liver fibrosis in hypoxia-inducible factor-1alpha-deficient mice. Am J Physiol Gastrointest Liver Physiol.

[20] Wen YA, Liu D, Zhou QY, Huang SF, Luo P, Xiang Y, Sun S, Luo D, Dong YF, Zhang LP (2011). Biliary intervention aggravates cholestatic liver injury, and induces hepatic inflammation, proliferation and fibrogenesis in BDL mice. Exp Toxicol Pathol.

[21] Falkowski O, An HJ, Ianus IA, Chiriboga L, Yee H, West AB, Theise ND (2003). Regeneration of hepatocyte ‘buds’ in cirrhosis from intrabiliary stem cells. J Hepatol.

[22] Duffield JS, Forbes SJ, Constandinou CM, Clay S, Partolina M, Vuthoori S, Wu S, Lang R, Iredale JP (2005). Selective depletion of macrophages reveals distinct, opposing roles during liver injury and repair. J Clin Invest.

[23] Copple BL, Kaska S, Wentling C (2012). Hypoxia-inducible factor activation in myeloid cells contributes to the development of liver fibrosis in cholestatic mice. J Pharmacol Exp Ther.

[24] Abe M, Koga H, Yoshida T, Masuda H, Iwamoto H, Sakata M, Hanada S, Nakamura T, Taniguchi E, Kawaguchi T, Yano H, Torimura T, Ueno T (2012). Hepatitis C virus core protein upregulates the expression of vascular endothelial growth factor via the nuclear factor-kappaB/hypoxia-inducible factor-1alpha axis under hypoxic conditions. Hepatol Res.

[25] Rosmorduc O, Wendum D, Corpechot C, Galy B, Sebbagh N, Raleigh J, Housset C, Poupon R (1999). Hepatocellular hypoxia-induced vascular endothelial growth factor expression and angiogenesis in experimental biliary cirrhosis. Am J Pathol.

[26] Corpechot C, Barbu V, Wendum D, Kinnman N, Rey C, Poupon R, Housset C, Rosmorduc O (2002). Hypoxia-induced VEGF and collagen I expressions are associated with angiogenesis and fibrogenesis in experimental cirrhosis. Hepatology.

[27] Hicklin DJ, Ellis LM (2005). Role of the vascular endothelial growth factor pathway in tumor growth and angiogenesis. J Clin Oncol.

[28] Taura K, De Minicis S, Seki E, Hatano E, Iwaisako K, Osterreicher CH, Kodama Y, Miura K, Ikai I, Uemoto S, Brenner DA (2008). Hepatic stellate cells secrete angiopoietin 1 that induces angiogenesis in liver fibrosis. Gastroenterology.

[29] Aleffi S, Petrai I, Bertolani C, Parola M, Colombatto S, Novo E, Vizzutti F, Anania FA, Milani S, Rombouts K, Laffi G, Pinzani M, Marra F (2005). Upregulation of proinflammatory and proangiogenic cytokines by leptin in human hepatic stellate cells. Hepatology.

[30] Kawashima R, Mochida S, Matsui A, YouLuTu ZY, Ishikawa K, Toshima K, Yamanobe F, Inao M, Ikeda H, Ohno A, Nagoshi S, Uede T, Fujiwara K (1999). Expression of osteopontin in Kupffer cells and hepatic macrophages and Stellate cells in rat liver after carbon tetrachloride intoxication: a possible factor for macrophage migration into hepatic necrotic areas. Biochem Biophys Res Commun.

[31] Shijubo N, Uede T, Kon S, Nagata M, Abe S (2000). Vascular endothelial growth factor and osteopontin in tumor biology. Crit Rev Oncog.

[32] Liu C, Yang Z, Wang L, Lu Y, Tang B, Miao H, Xu Q, Chen X (2015). Combination of sorafenib and gadolinium chloride (GdCl3) attenuates dimethylnitrosamine (DMN)-induced liver fibrosis in rats. BMC Gastroenterol.

[33] Medina J, Arroyo AG, Sanchez-Madrid F, Moreno-Otero R (2004). Angiogenesis in chronic inflammatory liver disease. Hepatology.

[34] Medina J, Sanz-Cameno P, Garcia-Buey L, Martin-Vilchez S, Lopez-Cabrera M, Moreno-Otero R (2005). Evidence of angiogenesis in primary biliary cirrhosis: an immunohistochemical descriptive study. J Hepatol.

[35] Chen CJ, Hsu MH, Huang LJ, Yamori T, Chung JG, Lee FY, Teng CM, Kuo SC (2008). Anticancer mechanisms of YC-1 in human lung cancer cell line, NCI-H226. Biochem Pharmacol.

